# Conscientious Non-objection in Intensive Care

**DOI:** 10.1017/S0963180116000700

**Published:** 2017-01

**Authors:** DOMINIC WILKINSON

**Keywords:** conscience, conscientious objection, refusal to treat/ethics, intensive care units/ethics, patient rights/ethics, professional autonomy, withdrawing treatment

## Abstract

Discussions of conscientious objection (CO) in healthcare often concentrate on objections to interventions that relate to reproduction, such as termination of pregnancy or contraception. Nevertheless, questions of conscience can arise in other areas of medicine. For example, the intensive care unit is a locus of ethically complex and contested decisions. Ethical debate about CO usually concentrates on the issue of whether physicians should be permitted to object to particular courses of treatment; whether CO should be accommodated. In this article, I focus on the question of how clinicians ought to act: should they provide or support a course of action that is contrary to their deeply held moral beliefs? I discuss two secular examples of potential CO in intensive care, and propose that clinicians should adopt a norm of conscientious non-objection (CNO). In the face of divergent values and practice, physicians should set aside their personal moral beliefs and not object to treatment that is legally and professionally accepted and provided by their peers. Although there may be reason to permit conscientious objections in healthcare, conscientious non-objection should be encouraged, taught, and supported.

## Conscientious Objection (CO)

Five to twenty percent of patients admitted to intensive care units (ICUs) die before discharge.^1^ The majority of deaths follow discussions and explicit treatment limitation decisions.^2^ Disagreements about treatment in intensive care occur commonly,^3^ are a frequent source of requests for ethics consultation,^4^ and may lead to court involvement.

For example, a large international survey of ICU clinicians found that 72 percent had experienced conflict in the previous week.^5^ Such conflicts were often perceived as “severe” and “dangerous,” and possibly harmful to quality of care and patient survival.^6^ Another large survey in European and Israeli ICUs found that a significant proportion of intensive care staff were participating in treatment that they regarded as “inappropriate.”^7^ The study suggested that on a given day, as many as 27 percent of nurses and doctors in intensive care were providing treatment to one or more patients that was contrary to his or her personal and professional beliefs.^8^

However, despite the frequency of moral distress, there are relatively few reports of actual CO in the ICU. One possible explanation is that in ICUs with large numbers of medical and nursing staff, it may be relatively easy to transfer care to another professional without a CO. A small survey of 66 pediatric and neonatal nurses found that 45 percent had acted contrary to a medical instruction, because of their conscience.^9^ Ten percent had covertly or overtly declined to follow an order, 10 percent had sought another nurse to take over care, and 17 percent had voiced dissent.^10^ Another possibility is that conscience may contribute to variation in medical decisions without an explicit objection. There is significant physician-related variability in end-of-life decisions in ICUs.^11^ For example, a recent study using simulated patients found strikingly low agreement among physicians on whether they would admit an elderly patient to intensive care.^12^ There is considerable variation in rates of withholding and withdrawal of life-sustaining treatment among ICUs in the same region.^13^ In one study within a single ICU, there was a 15-fold variation between clinicians in the rates of limitation of treatment; decisions to limit treatment were more strongly related to which physician was the attending consultant than to patient comorbidity and diagnostic category.^14^

A recent guideline from the American Thoracic Society (ATS) analyzes the question of whether institutions should accommodate professionals who have a conscientious objection to treatment plans in the ICU, and sets out recommendations for institutional policies for CO.^15^ However, a separate ethical question is the focus of this article: How should the professional respond to conflicts between their conscience and potential treatment decisions?

First, it will be helpful to be clear what we are discussing. Here is one definition of medical conscientious objection.^16^

*Conscientious objection:* A considered decision by a medical professional to *not provide* a legal and professionally accepted medical course of action requested by or on behalf of a patient *on the basis of a personal belief* that this action would be morally wrong*.*

Discussion of CO often focuses on religious objections to particular treatment. Sources of moral distress in the ICU, however, do not necessarily arise from religious objections.

### Controversial Provision of Treatment: Conscientious Objection to Treatment Perceived as Futile

The most frequent reason for ICU clinicians judging treatment to be “inappropriate” is a belief that the patient is receiving excessive or unbeneficial medical treatment.^17^ In extreme cases this can lead to physicians and nurses declining to care for that patient. For example, in 2008, a dispute arose over the medical care of Samuel Golubchuk, an 84-year-old man with multiple organ failure and brain damage.^18^ Medical staff wished to discontinue treatment, whereas Mr Golubchuk’s family wished treatment to continue. It was reported that three physicians in the ICU refused to care for the patient because they regarded his treatment as unethical.^19^

It is important to note that some authors have argued that refusal to provide treatment on grounds of futility is not a true CO (because it is based on professional norms/standards rather than personal values).^20^ However, the boundary between clinical disagreement and moral disagreement is often blurred. A determination that treatment is futile or unbeneficial can clearly be influenced by personal values,^21^ and, as is highlighted in this case, can lead to individuals declining to be involved in treatment. The ATS guideline includes moral objections to treatment (on the grounds of perceived futility) as a form of CO.^22^

### Controversial Nonprovision of Treatment: CO to Premature Limitation of Treatment/Palliative Care

The opposite ethical concern can arise when physicians or nurses have ethical objections to limitations of treatment.^23^ In a survey of 690 European intensive care professionals, approximately 43 percent of physicians and 53 percent of nurses indicated that they would go against a patient’s wish to refuse treatment if they felt that the patient would benefit from it.^24^ It is rare that such an objection would be absolute; physicians or nurses may accept limitation of treatment in some cases, but regard the outcome in a particular case as too uncertain (or too positive) to be comfortable forgoing support. It is of note that in the United States, 5 percent of physicians indicated an in-principle objection to withdrawal of life support.^25^ Objections of this nature sometimes impact on patients. In 2002, the United Kingdom courts heard the case of Miss B, a 45-year-old woman with long-standing spinal cord problems, who requested to be disconnected from the ventilator after she became quadriplegic.^26^ Intensive care physicians declined her request, although she had been assessed as competent by psychiatrists. They were prepared to institute a gradual process of reducing breathing support, and wished to give Miss B a further period of rehabilitation to see if she changed her mind. However, after more than a year of receiving treatment against her wishes, Miss B eventually sought (and obtained) court support to be allowed to die.^27^

Clinicians in intensive care may or may not refer to their concerns using the language of “conscience” (they may refer to treatment options as “not clinically indicated” or “unethical”). Their concerns may be specific to particular cases, or they may be in-principle concerns about particular treatment options in intensive care. Regardless, the ethical question is the same: should they, or should they not object?

## Conscientious Non-objection

There are a number of reasons why institutions should potentially be prepared to accommodate objections to perceived futile treatment or to premature limitation of treatment.^28^ The ATS statement suggests that this would protect clinicians’ autonomy and moral integrity, and could improve quality of care.^29^ I accept that accommodation may be necessary. However, in cases such as the ones described, I argue that the individual clinician *should* in conscience support the family’s and patient’s request, notwithstanding their qualms. Call this conscientious non-objection:

*Conscientious non-objection*: A considered decision by a medical professional to *provide* a legal and professionally accepted medical course of action requested by or on behalf of a patient *despite a personal belief* that this action would be morally wrong.

The term “conscientious non-objection” here might be thought to be an oxymoron. I employ the term deliberately to take advantage of the dual implication that the physician is not-objecting (despite that physician’s conscience), and is also acting conscientiously in doing so. An alternative term for this phenomenon might be “professional non-objection,” which would allude to the professional nature of the disagreement, as well as to one reason why the individual ought not to object.^30^ (None of the subsequent argument is dependent on the specific terminology)

CNO describes a decision not to object, despite the call of conscience. Why should clinicians act contrary to their moral beliefs? Here (in the next section) are three potential reasons.

## Arguments in Favor of CNO

### Autonomy

First, CNO respects patients’ rights to access treatment options that have been deemed legally permissible, and endorsed by members of the profession. This argument is based on respect for the patient autonomy, and patients’ freedom to make decisions about their own lives. Autonomy presents a strong justification for allowing patients to refuse treatment, even where the physician judges that the treatment would be in their best interests. This might ground a case for CNO in the context of limitation of treatment/palliative care. For example, two clinicians described their personal response to a case of ventilator disconnection in a paralyzed man, similar to the Miss B case.^31^ One of the physicians had strong pro-life beliefs, and clearly felt deeply conflicted about the decision to remove the ventilator. However, he was also reported to support individuals’ right to refuse treatment.^32^

Autonomy might not provide as strong an argument in favor of providing treatment that the patient requests. Because autonomy is often construed in negative terms; that is, with regard to refusing treatments, patients are not usually thought to have a right to demand treatment.^33^ However, in situations in which other physicians would provide that same requested treatment, a physician’s refusal does appear to compromise the patient’s autonomy.

### Justice

Second, CNO addresses or reduces one potentially unjust feature of some medical decisionmaking: the apparent variability of physician decision making, and influence of personal values. I noted significant variation between physicians in intensive care in their end-of life decisions. This appears to affect provision of intensive care, as well as withdrawal of intensive care. Such variability can mean that whether or not patients are able to access admission to intensive care, or (conversely) the option of palliative care, depends on which physician happens to be on duty.^34^ However, the patient or the patient’s family will often be completely unaware that such variation exists, and may not share with the physician the values that have led to the decision. This variation appears arbitrary and unjust. If clinicians know that their peers would offer a particular treatment option, they should arguably also offer that treatment option, or at the very least make the patient/surrogate aware of the different views that exist.^35^

### Moral Uncertainty

Third, CNO is justified on the grounds of moral uncertainty. When we make a moral judgment that a particular course of action would be right (or wrong), we should take into account the possibility of error.^36^ Differing views in the cases described highlight that moral uncertainty exists. For individual clinicians, the fact that their assessments of the ethics differ both from society’s (as evidenced by this course being lawful) and from those of peers (who are prepared to provide a particular service) means that they should take seriously the possibility that their own view is mistaken. What they should do given this uncertainty is more difficult. There are different types and levels of moral uncertainty, as well as different theoretical approaches to dealing with such uncertainty. It is difficult to know how to evaluate the probability of error, or how much weight to give to different possible outcomes (assuming that a particular moral judgment is correct). However, plausibly in the case of genuine moral uncertainty, medical decisions should be guided by the values of the patient, not by the values of the provider. Clinicians should be prepared to set aside their own moral assessment and not object.^37^

## CNO and the “Dissensus Approach”

The above-mentioned arguments in favor of CNO parallel arguments that I have made previously in support of a so-called “dissensus” approach to decisions about withdrawal of life-sustaining treatment.^38^ Professional guidelines promote the idea that these very serious decisions should be based on professional *consensus*; that is that agreement of a majority of professionals is a necessary condition for the ethical permissibility of a decision leading to the death of the patient. However, I argued that although agreement was desirable, it was not necessary. On the contrary, professional disagreement was indicative of varying ethical assessments and moral uncertainty; it pointed to the need for such decisions to be based on the values of the patient, rather than those of individual (or even the majority of) healthcare providers. In that article I proposed that the permissibility of withdrawing treatment depended on there being at least one member of the treating team who was prepared (after adequate reflection and discussion) to support this course, and who would take over the care of the patient if necessary. This would potentially allow an individual clinician or multiple clinicians to conscientiously object if they chose without adversely affecting the availability of the option for the patient.

However, based on the abovementioned arguments, my claim here is stronger: clinicians *should not* object, despite their personal beliefs. Why make this normative claim, if the patient would still be able to access withdrawal of treatment? One reason is that by requiring a peer to take over the care of the patient, the objector places a significant burden on a colleague.^39^ This includes the time and energy involved in caring for an additional patient, as well as the additional emotional burden of providing end-of-life care.^40^ It also potentially places an additional burden on the patient and family, where the transfer of care disrupts an existing care relationship, or is interpreted by the patient/family as a form of implicit moral censure or disapprobation.^41^

The dissensus approach was described in relation to treatment limitation. It therefore applies to cases in the section entited “Controversial Nonprovision of Treatment,” in which there was disagreement about limitation of treatment and provision of palliative care. This approach can also be applied, however, to cases of controversial provision of treatment. For example, in the Samuel Golubchuk case, a number of healthcare professionals objected to providing medical treatment, whereas others were prepared to continue treatment pending court assessment. Given apparent dissensus, it appears prima facie to have been permissible to continue to provide intensive care to Mr. Golubchuk. Moreover, the abovementioned arguments suggest that the individual professionals *should not* have conscientiously objected to his treatment.

## Arguments Against CNO

I have proposed that patient autonomy, justice, and moral uncertainty provide strong reasons why healthcare professionals in intensive care should not exercise their right to CO. There are several potential counterarguments to this proposal. I will discuss five of them in the following subsections.

### Incoherence

One potential concern is that CNO is incoherent or contradictory. I have argued that an individual clinician *ought* to take a course of action that they sincerely believe they *ought not* to take. A critic might ask whether it makes any sense for a clinician in the case example to believe simultaneously that they should and should not provide treatment to Mr. Golobchuk.

There are several potential responses to this concern. First, the nature of any true moral dilemma is that there is a conflict between moral requirements. The existence of contradictory norms is not surprising in these cases. Second, one way of characterizing the two requirements is that individuals have a *pro tanto* reason not to act in a certain way (for example, not to provide ostensibly futile treatment), whereas they have an *all-things-considered* reason to acquiesce and provide the requested treatment. Does this avoid incoherence? It may be that the individual clinician strongly believes that he or she has an all-things-considered reason not to withdraw treatment or to provide futile treatment. In that case, we could see the argument in favor of CNO as providing something that the professional should consider seriously prior to formally objecting. It provides some additional considerations that the clinician may not have factored in to an all-things-considered judgment.

### Moral Distress

I noted at the start of this article that one consequence of the difficult decisions encountered in intensive care is the high rate of moral distress. It might be thought that CO provides a mechanism for alleviating moral distress (individual providers may choose not to participate in decisions that they would find distressing), whereas conversely, CNO would potentially increase distress. Should the medical profession therefore be more encouraging of objection?

Whether a particular policy or norm would lead to more or less distress among professionals is an empirical question. There are no existing data (to my knowledge) to assess whether promoting CNO would lead to more moral distress among staff. If so, there would then be an ethical question to answer: Whether this distress outweighed the reasons in favor of CNO. However, it is not necessarily the case that endorsing or promoting CNO would increase distress, and it is possible that it would reduce it. Moral distress is often said to reflect a sense of disequilibrium resulting from being unable to take a course of action that one recognizes as ethically appropriate.^42^ The arguments in favor of non-objection encourage professionals to be cognizant of their peers, of the variation in views that are held, and of the potential moral uncertainty at stake in such assessments. This would provide them with positive reasons to believe that they are doing the right thing in not objecting, and potentially help relieve their sense of discomfort.

### Religious Objection

The examples I gave in this article were based on secular objections to provision or limitation of treatment in intensive care. Some may feel, however, that this focuses on easier cases of non-objection; religious cases of CO potentially pose more of a challenge. When a professional has an in-principle objection to a particular practice on the basis of core religious beliefs, it may be harder to imagine that that person will be swayed by arguments such as those outlined previously.

For example, the argument from autonomy will not necessarily convince a health professional who believes that the act in question is subject to an absolute religious prohibition. Likewise, the argument from moral uncertainty relies on giving some credence to the possibility that alternative moral assessments are possibly true. However, at least some religious adherents will believe strongly that their particular moral judgment is objectively morally correct, and that any alternative viewpoint is mistaken.

However, the arguments for CNO arguably still apply to religiously motivated concerns about provision or non-provision of treatment. The value of patient autonomy does not depend on the professional agreeing with the patient. In fact, the contrary is the case: Professionals’ support for patient autonomy only counts when they respect a patient’s choice despite regarding it as the wrong choice. In particular, patients have an absolute right to refuse treatment. It would be unreasonable to impose treatment on the patient that the patient does not want, on the basis of religious values that the patient does not share. Variation among clinicians in the options that they would offer to patients does not become justified when physicians have varying religious beliefs. On the contrary, diversity of religious beliefs ought to lead clinicians to be cautious about the way in which their personal values are influencing the professional judgments that they are making.

The question of moral uncertainty raises the issue of the epistemology of disagreement in the face of religious diversity.^43^ There are different views about how a religious adherent should respond to evidence of interfaith and intrafaith differences in normative judgments. Some have argued that humility in the face of religious diversity should lead to reduced confidence in one’s own belief and tolerance of the views of others.^44^ Others maintain that the lack of common ground for resolving disputes means that it is not irrational for individuals to remain committed to the truth of their religious beliefs to the exclusion of other beliefs.^45^ It is beyond the scope of this article to resolve those questions. However, separate from those epistemic questions is the issue of religious pluralism in democratic societies. Most democracies without state religions have explicit commitments to tolerance of diverse religious views. In societies such as the United States, Australia, and the United Kingdom, there is strong political and sometimes constitutional support for freedom of religion. Although such freedom may well provide a justification for *permitting* religious-based CO, it would also support the idea that those acting in public office, or in public service (such as healthcare) should not impose their personal religious views on those whom they are serving. It thus provides an additional argument in support of CNO.

### Resource Limitations

One potential argument against CNO relates specifically to controversial requests for continued treatment, and is highlighted by the case of Samuel Golobchuk. In cases such as this, continued treatment may consume limited healthcare resources and may harm other patients (because they are unable to access treatment).^46^ If a clinician’s refusal is based on this sort of concern, should objection (rather than non-objection) be encouraged?

A full discussion of the role of resources in CO is beyond the scope of this article. Finite resources might provide a limit to medical decisions either in concordance with, or in opposition to, patients’ wishes. They may certainly provide a reason to decline a patient’s request for continued treatment.

However, resource-based treatment refusals ought to be based on a fair process that is transparent, consistent, based on relevant reasons, and, ideally, based on clearly articulated policy.^47^ They should not to be based on the conscience and values of the individual clinician – rather on the values and deliberation of society.

### Unjust Norms

I have argued that a physician ought to provide a medical treatment option that is professionally and legally endorsed despite that physician’s personal moral beliefs that this would be wrong. However, there may be conceivable situations in which a physician has reason to question whether the relevant ethical and legal norms are themselves just. There are plenty of historical examples in which particular medical practices were generally endorsed, but which, in retrospect, were highly ethically problematic.

We could imagine, for example, a country that has strict religious or cultural norms against withdrawal of treatment, and against the wishes of a patient such as Miss B who strongly desires that treatment not be provided. Should the physician conscientiously object to the norm against treatment withdrawal? Alternatively, it may be that a particular health system has institutional policies that discriminate against patients with a particular illness or disability, and would deny such patients access to intensive care. In these cases, should a physician listen to his or her conscience and admit the patient to intensive care or decline admission?

It is important to note here that if withdrawal of treatment (in the first case) or admission to intensive care (in the second) is performed at the request, or for the benefit, of the patient, neither of these circumstances would fit with the definition of CNO that I proposed. The patient’s wishes are contrary to the norms of society. The arguments I outlined from autonomy, justice, and moral uncertainty would support objection rather than non-objection in such cases.

In other circumstances, in which a patient’s wishes are consonant with professional and legal norms that the physician believes are morally wrong or unjust, the ethically appropriate course is not to object to the treatment requested, but rather to campaign publicly for a change in those norms.^48^

## Conclusions

In this article, I have argued that health professionals *should* sometimes provide medical treatment options that they personally find morally troublesome. They *should not* object to professionally accepted and legal courses of medical treatment that have been requested by or on behalf of a patient.

I have defended CNO in the context of intensive care, and applied it both to controversial decisions to provide or continue life-sustaining treatment, as well as to controversial decisions to limit treatment or provide end-of-life care. Although I have not explored such examples, the arguments in favor of CNO would also apply to treatment options outside intensive care, including reproductive decisions that are often the focus of CO.

I have drawn on secular examples of potential objection, because in the ICU, explicit objection to particular options, as well as variable views about individual cases, are not necessarily motivated by religious views. However, where religion does underlie concern, the arguments from autonomy, justice, and moral uncertainty would still favor non-objection.

None of the arguments presented here mean that medical professionals should be forced to provide treatments that are in conflict with their deeply held views. There are a number of reasons to accommodate professional CO, as long as there are adequate safeguards to protect patients’ well-being and access to treatment. However, we should encourage health professionals to be ethically humble and tolerant. They should in conscience, and sometimes despite their conscience, inform patients about, facilitate, and provide professionally accepted and legal medical treatment options.

## Notes

1. ChowS, ChowR, PopovicM, LamM, PopovicM, MerrickJ, et al A selected review of the mortality rates of neonatal intensive care units. Frontiers in Public Health 2015;3:225.2650104910.3389/fpubh.2015.00225PMC4595739

2. SprungCL, CohenSL, SjokvistP, BarasM, BulowHH, HovilehtoS, et al End-of-life practices in European intensive care units: The Ethicus Study. JAMA 2003;290(6):790–7;1291543210.1001/jama.290.6.790 FontanaMS, FarrellC, GauvinF, LacroixJ, JanvierA. Modes of death in pediatrics: differences in the ethical approach in neonatal and pediatric patients. Journal of Pediatrics 2013;162(6):1107–11.2331268510.1016/j.jpeds.2012.12.008

3. AzoulayE, TimsitJF, SprungCL, SoaresM, RusinovaK, LafabrieA, et al Prevalence and factors of intensive care unit conflicts: the conflicus study. American Journal of Respiratory and Critical Care Medicine 2009;180(9):853–60.1964404910.1164/rccm.200810-1614OC

4. TapperEB, VerclerCJ, CruzeD, SexsonW. Ethics consultation at a large urban public teaching hospital. Mayo Clinic Proceedings 2010;85(5):433–8.2043583610.4065/mcp.2009.0324PMC2861972

5. See note 3, Azoulay et al. 2009.

6. See note 3, Azoulay et al. 2009.

7. Piers RD, Azoulay E, Ricou B, Dekeyser Ganz F, Decruyenaere J, Max A, et al. Perceptions of appropriateness of care among European and Israeli intensive care unit nurses and physicians. JAMA 2011;306(24):2694–703.

8. See note 7, Piers et al. 2011.

9. CatlinA, ArmigoC, VolatD, ValeE, HadleyMA, GongW, et al Conscientious objection: A potential neonatal nursing response to care orders that cause suffering at the end of life? Study of a concept. Neonatal Network 2008;27(2):101–8.1843196410.1891/0730-0832.27.2.101

10. See note 9, Catlin et al. 2008.

11. WilkinsonDJ, TruogRD. The luck of the draw: Physician-related variability in end-of-life decision-making in intensive care. Intensive Care Medicine 2013;39(6):1128–32.2343595110.1007/s00134-013-2871-6

12. Garrouste-Orgeas M, Tabah A, Vesin A, Philippart F, Kpodji A, Bruel C, et al. The ETHICA study (part II): Simulation study of determinants and variability of ICU physician decisions in patients aged 80 or over. *Intensive Care Medicine* 2013;39(9):1574–83.

13. MarkNM, RaynerSG, LeeNJ, CurtisJR. Global variability in withholding and withdrawal of life-sustaining treatment in the intensive care unit: A systematic review. Intensive Care Medicine 2015;41:1572–85.2590418310.1007/s00134-015-3810-5

14. GarlandA, ConnorsAF. Physicians’ influence over decisions to forego life support. Journal of Palliative Medicine 2007;10(6):1298–305.1809580810.1089/jpm.2007.0061

15. Lewis-NewbyM, WicclairM, PopeT, RushtonC, CurlinF, DiekemaD, et al An official American Thoracic Society policy statement: managing conscientious objections in intensive care medicine. American Journal of Respiratory and Critical Care Medicine 2015;191(2):219–27.2559015510.1164/rccm.201410-1916ST

16. This definition has some overlap with one provided by Mark Wicclair. (See Wicclair MR. *Conscientious Objection in Health Care: An Ethical Analysis*. Cambridge: Cambridge University Press; 2011, at.1–4).Wicclair notes two necessary components: (1) a refusal by a health professional to perform or provide a legally and professionally accepted good or service, and (2) that the individual justifies refusal on the basis of core moral beliefs. COs are possible in other areas of life, (for example objections to military service) but are beyond the scope of this article. I have restricted the scope of CO here to courses of treatment requested by or on behalf of the patient. This excludes questions about CO to physician involvement in torture or capital punishment (neither of which are relevant in intensive care).

17. See note 7, Piers et al. 2011.

18. JotkowitzA, GlickS, ZivotofskyAZ. The case of Samuel Golubchuk and the right to live. American Journal of Bioethics 2010;10(3):50–3.10.1080/1526516090358170020229421

19. Winnipeg Free Press. Winnipeg hospitals divert key resources to care for dying man. Canada.com June 20, 2008; available at http://www.canada.com/topics/news/national/story.html?id=9c5d6629-64f2-4b57-86fc-496be65c2664 (last accessed 19 Jan 2016). In this case there was a reported religious motivation for the request for treatment, but no religious reason was apparent for the clinicians who were conscientiously objecting.

20. See note 16, Wicclair 2011, at 7–8.

21. See note 11, Wilkinson, Truog 2013.

22. See note 15, Lewis-Newby et al. 2015. Case 3 in the American Thoracic Society document (at 223) represents an example of controversial provision of treatment.

23. Wicclair MR. Conscientious objection in medicine. *Bioethics* 2000;14(3):205–27, at 207. Case 4 in in the American Thoracic Society document (at 223) also represents an example of this kind.

24. BulowHH, SprungCL, BarasM, CarmelS, SvantessonM, BenbenishtyJ, et al Are religion and religiosity important to end-of-life decisions and patient autonomy in the ICU? The Ethicatt study. Intensive Care Medicine 2012;38(7):1126–33.2252707010.1007/s00134-012-2554-8

25. Curlin FA, Nwodim C, Vance JL, Chin MH, Lantos JD. To die, to sleep: US physicians’ religious and other objections to physician-assisted suicide, terminal sedation, and withdrawal of life support. *American Journal of Hospice and Palliative Care* 2008;25(2):112–20.

26. Dyer C. We felt we had killed him. *The Guardian* March 13, 2002; available at http://www.theguardian.com/society/2002/mar/13/health.law (last accessed 20 Jan 2016)

27. See note 26, Dyer 2002.

28. WhiteDB, BrodyB. Would accommodating some conscientious objections by physicians promote quality in medical care? JAMA 2011;305(17):1804–5.2154042510.1001/jama.2011.575

29. See note 15, Lewis-Newby et al. 2015.

30. I am grateful to an anonymous reviewer for this point.

31. EdwardsMJ, TolleSW. Disconnecting a ventilator at the request of a patient who knows he will then die: The doctor’s anguish. Annals of Internal Medicine 1992;117(3):254–6.161622110.7326/0003-4819-117-3-254

32. See note 26, Dyer 2002.

33. ParisJJ. Autonomy does not confer sovereignty on the patient: A commentary on the Golubchuk case. American Journal of Bioethics 2010;10(3):54–6.10.1080/1526516090358191620229422

34. See note 11 Wilkinson, Truog 2013.

35. WilkinsonD, TruogR, SavulescuJ. In favour of medical dissensus: Why we should agree to disagree about end-of-life decisions. Bioethics 2016;30(2):109–8.2590839810.1111/bioe.12162PMC4864446

36. LockhartT. Moral Uncertainty and Its Consequences. New York, Oxford: Oxford University Press; 2000.

37. Although moral uncertainty suggests that an individual clinician should decide not to object, it also plausibly means that society should *permit* or accommodate objection.

38. See note 35, Wilkinson et al. 2015.

39. MagelssenM. When should conscientious objection be accepted? Journal of Medical Ethics 2012;38(1):18–21.2169023010.1136/jme.2011.043646

40. I do not have any empirical data here to quantify this burden. My personal experience, however, is that providing high quality care at the end of life involves a substantial commitment of time on the part of senior medical staff as well as nursing staff. This includes time spent with the patient and with the family discussing treatment options and coming to a decision to limit treatment, as well as subsequently ensuring that their needs are addressed. In practice, such patients can require far more intensive personal involvement than other patients in intensive care, with significant physical and emotional consequences for professionals. See Kirby E, Broom A, Good P. The role and significance of nurses in managing transitions to palliative care: a qualitative study. *BMJ Open* 2014;4(9):e006026; Lee KJ, Dupree CY. Staff experiences with end-of-life care in the pediatric intensive care unit. *Journal of Palliative Medicine* 2008;11(7):986–90.

41. See note 39, Magelssen 2012.

42. MobleyMJ, RadyMY, VerheijdeJL, PatelB, LarsonJS. The relationship between moral distress and perception of futile care in the critical care unit. Intensive and Critical Care Nursing 2007;23(5):256–63.1768146810.1016/j.iccn.2007.03.011

43. ChristensenD. Disagreement as evidence: The epistemology of controversy. Philosophy Compass 2009;4(5):756–67.

44. QuinnP. On religious diversity and tolerance. Daedalus 2005;134(1):136–9.

45. AlstonW. Religious diversity and perceptual knowledge of God. Faith and Philosophy 1988;5(4):433–48.

46. WilkinsonDJC, SavulescuJ. Knowing when to stop: Futility in the ICU. Current Opinion in Anesthesiology 2011;24(2):160–5.2129326710.1097/ACO.0b013e328343c5afPMC3252683

47. DanielsN. Justice, health, and healthcare. American Journal of Bioethics 2001;1(2):2–16.10.1162/15265160130016883411951872

48. SavulescuJ. Conscientious objection in medicine. BMJ 2006;332(7536):294–7.1645573310.1136/bmj.332.7536.294PMC1360408

